# Notices, Reviews, &c.

**Published:** 1859-12

**Authors:** 


### NOTICES, REVIEWS, &c.

Our editorial brethren are respectfully requested to give notice that the *Journal of Health* begins its Seventh Volume with January, and that now is a good time to subscribe, sending their subscriptions to Dr. W. W. Hall, New York, Editor.

The Messrs. Fowler & Wells, 308 Broadway, New York, have just published *Hints Towards Physical Perfection, or the Philosophy of Human Beauty;* showing how to retain and acquire bodily symmetry, health and long life. By Dr. H. Jacques, pp. 244, 75 cts., 1859. This book contains much that is useful, true and safe.

"*The Right Word in the Right Place*," 214, 18mo., by the same publishers; 50 cents. Being a pocket dictionary and reference book, about writing for the press, punctuation, proof reading, synonyms, technical terms, mottos, foreign words and phrases. It is one of the most unexceptionably useful publications ever issued by that house.

*The Christian Review*, (Baptist,) $3.00 a year. Edited by Rev. Drs. E. G. Robinson and V. R. Hotchkins, and published by Sheldon & Co., at 115 Nassau street, New York. It enters its twenty-fifth volume, and is one of the very ablest religious reviews published in this country.

Leonard Scott & Co., 79 Fulton street, New York, republish, promptly and in beautiful style, for $10.00 a year, *Blackwood's Monthly Magazine* and the four Quarterlies, that is, the *London*, *Edinburgh*, *Westminster* and *North British*, or separately, $3.00 a year.

*The Pacific Expositor*, edited by Rev. W. A. Scott, D. D., of San Francisco, monthly, $3.00 a year, has, as we perceive from our exchanges, met with a wide and cordial reception. It richly merits, and we trust it will receive a prompt and wide patronage.

*Our Daughters.* Mrs. T. P. Smith, the accomplished Principal of Mystic Hall Seminary, near Boston, Mass., has assumed the direction of the Washington (D. C.) Female Institute. Her capability and energy will achieve success in her new enterprize, as it has done before in New England.

*Buttermilk.* Some of the medical journals are advocating the free use of buttermilk, as having a large efficiency in enabling persons to grow old without that stiffness of joint and limb so common to the aged, and that it is greatly promotive of the general health of all. We can testify to two things, that we are very healthy, and that we love and use buttermilk daily from the New Jersey and Rockland County Milk Association, in Tenth street, near Broadway; but we can't conscientiously or wisely say, whether buttermilk gives health or not, for we were well enough before we commenced drinking it, and are well enough still. We can, however, say this, that it does not appear to be hurtful, which is more than can be asserted of beer, gin, wine, brandy, whisky, coffee, tea, tobacco and swill milk.

